# Symptom Network Analysis in a Large Sample of Children and Adults with a Chronic Tic Disorder

**DOI:** 10.1002/mdc3.14167

**Published:** 2024-07-25

**Authors:** Caroline Garcia Forlim, Valerie Brandt, Ewgeni Jakubovski, Christos Ganos, Simone Kühn, Kirsten Müller‐Vahl

**Affiliations:** ^1^ Neuronal Plasticity Working Group, Department of Psychiatry and Psychotherapy University Medical Center Hamburg‐Eppendorf Hamburg Germany; ^2^ Center for Environmental Neuroscience Max Planck Institute for Human Development Berlin Germany; ^3^ School of Psychology, Centre for Innovation in Mental Health University of Southampton Southampton UK; ^4^ Clinic of Psychiatry, Social Psychiatry and Psychotherapy Hannover Medical School Hannover Germany; ^5^ Movement Disorder Clinic, Edmond J. Safra Program in Parkinson's Disease, Division of Neurology University of Toronto, Toronto Western Hospital Toronto Ontario Canada

**Keywords:** chronic tic disorder, network theory, symptom network

## Abstract

**Background:**

Chronic tic disorders (CTD) are multifaceted disorders characterized by multiple motor and/or vocal tics. They are often associated with complex tics including echophenomena, paliphenomena, and coprophenomena as well as psychiatric comorbidities such as attention deficit/hyperactivity disorder (ADHD) and obsessive‐compulsive disorder (OCD).

**Objectives:**

Our goal was to uncover the inter‐relational structure of CTD and comorbid symptoms in children and adults and to understand changes in symptom structure across development.

**Methods:**

We used network and graph analyses to uncover the structure of association of symptoms in childhood/adolescence (n = 529) and adulthood (n = 503) and how this structure might change from childhood to adulthood, pinpointing core symptoms as a main target for interventions.

**Results:**

The analysis yielded core symptom networks in young and adult patients with CTD including complex tics and tic‐related phenomena as well as touching people and objects. Core symptoms in childhood also included ADHD symptoms, whereas core symptoms in adults included symptoms of OCD instead. Interestingly, self‐injurious behavior did not play a core role in the young CTD network, but became one of the central symptoms in adults with CDT. In addition, we found strong connections between complex motor and vocal tics as well as echolalia and echopraxia.

**Conclusions:**

Next to other complex tics, echophenomena, paliphenomena, and coprophenomena can be regarded core symptoms of CTD. ADHD symptoms are closely related to CTD in childhood, whereas symptoms of OCD and self‐injurious behavior are closely associated with CTD in adults. Our results suggest that a differentiation between motor and vocal tics is somewhat arbitrary.

Tourette syndrome (TS) is a chronic tic disorder (CTD) that is characterized by multiple motor and vocal tics^1^ and affects ~0.5% of the population.^2^ Epidemiological data show that up to 90% of CTD patients suffer from comorbidities, most often obsessive‐compulsive disorder (OCD) and attention deficit/hyperactivity disorder (ADHD).^3^ Complex phenomena, such as echophenomena, coprophenomena, self‐injurious behavior (SIB), and aggressive behavior^4^, ^5^ are currently considered complex tics; however, they may also be related to comorbidities. In fact, diagnostic understanding of CTD and TS is shifting toward viewing these disorders within a spectrum, with transient tic disorder at one end of the spectrum and a complex presentation of multiple complex tics, including echo phenomena and coprophenomena, plus comorbidities at the other end of the spectrum.^6^

Approximately 90% of adults with a tic disorder report that they experience an uncomfortable sensation before executing a tic and that this sensation subsides once the tic is executed.^7^, ^8^, ^9^, ^10^, ^11^ This increase and decrease of premonitory urges around single tics or, more commonly, bouts of tics, has been shown experimentally in a majority of patients,^12^, ^13^ whereas a minority experiences different urge patterns.^13^ It has been assumed that premonitory urges may be a prerequisite for tic suppression, but experimental data could not confirm this assumption.^14^, ^15^ Nevertheless, premonitory urges are used in one of the most common behavioral interventions, habit reversal training, to signal oncoming tics.^16^, ^17^

The presentation of tics, tic‐related phenomena and comorbidities can be highly diverse in TS. Different symptoms of TS have previously been associated with specific comorbidities, for instance, the “non‐just‐right” feeling has been associated with comorbid OCD,^18^ whereas tic suppression ability has not been linked to comorbidities.^19^, ^20^ Several studies have used relatively large datasets to cluster symptoms in TS using factor analysis.^21^, ^22^ The results suggest a division between simple and complex symptoms in patients with TS, whereas OCD was not clearly related to one factor.^21^ Network analysis is a novel way to investigate symptoms,^23^ with the advantage that it can uncover complex associations in the inter‐relational structure of clinical symptoms.^24^

In the present analysis, a network approach was taken, including tic disorder symptoms and common comorbidities, and assessed in a large population of TS/CTD patients. The aim of the study was to establish how different phenomena in CTD are related to each other, to comorbid diagnoses, and which symptoms are central to the disorder. For that, we make use of graph measures where nodes represent symptoms and their links represent relationships between pairs of symptoms.

## Methods

### Participants

In total, n = 1032 patients were included. The sample was divided by age to assess how the symptom network changes from childhood to adulthood. The young sample included n = 529 children and adolescents (mean age = 10.9, standard deviation [SD] = 3.2, range = 4–17 years, female n = 108, 20.3%), the adult sample included n = 503 adults (mean age = 31.5, SD = 10.7, range = 18–72 years, female n = 118, 24.3%). For binary data, sample sizes of 250 to 350 people are sufficient to produce accurate cross‐sectional network estimations.^25^

Data was collected at the Hannover Medical School, the largest TS outpatient center in Germany. All patients were clinically evaluated and diagnosed by a TS specialist (K.M.V.) Data was extracted from medical records between the years 1995 to 2013. Records included semi‐structured interviews regarding tics and comorbidities, based on the National Hospital Interview Schedule.^26^ TS/CTD were diagnosed based on Diagnostic and Statistical Manual of Mental Disorders (DSM) criteria,^1^, ^27^ tic severity was assessed using the Shapiro Tourette Syndrome Severity Scale.^28^, ^29^ Lifetime occurrence of the following complex TS‐related phenomena was assessed as present (1) or absent (0): echopraxia, echolalia, palilalia, copropraxia, coprolalia, premonitory urges, and the ability to suppress tics. In addition, lifetime prevalence of the following comorbid symptoms and disorders was assessed clinically: obsessions, compulsions, touching people, touching objects, hyperactivity, inattention, impulsivity, anxiety disorder (including phobias, generalized anxiety, and panic disorder), major depression, substance use disorder (nicotine, alcohol, food, gambling, medication, and recreational drugs), sleep disorder, SIB, and aggression against others (family members, other people). Only n = 3 adolescents reported substance use disorder (n = 2 nicotine, n = 1 alcohol), but it was still included in the young CTD network, to keep it parallel to the adult network.

Descriptive information about the database has been published previously.^9^ Written informed consent was not required for this secondary data analysis.

### Networks

To calculate a network, nodes and links need to be inferred. Nodes represent individual symptoms in the patient population, and the strength of the links represent the correlation strength between pairs of symptoms given by mutual information.^30^ Our network comprises a binary series of symptoms (1 = present; 0 = absent) and tic‐related phenomena of CTD where 23 variables were selected based on availability in the dataset. The 23 variables include: simple vocal tics, complex motor tics, complex vocal tics, coprolalia, palilalia, echolalia, copropraxia, echopraxia, touching objects, touching people, SIB, obsessions, compulsions, premonitory urges, suppressibility of tics, anxiety, depression, substance use disorder, hyperactivity, impulsivity, inattention, aggression toward others, and sleep disorder. Simple motor tics had to be excluded from the analysis because all but one patient had simple motor tics. The binary data is coded as 1 (symptom present) and 0 (symptom absent). Because our data is naturally discrete and binary, we used the simple estimation of mutual information with the equation below:

$$\text{I}\left(\text{X}\text{;}\text{Y}\right)\text{=}\text{H}\left(\text{X}\right)\text{−}\text{H}\left(\text{X|Y}\right)\text{=}\sum_{x\text{∈}\text{X}\text{,}y\text{∈}\text{Y}}\text{p}\left(x,y\right)\log_{\text{2}}\left(\frac{\text{p}\left(x,y\right)}{\text{p}\left(x\right)\text{p}\left(y\right)}\right)$$

$$\text{I}\left(\text{X}\text{;}\text{Y}\right)\text{≤}\text{H}\left(\text{X}\right)$$

where I(X: Y)is the mutual information between X and Y. Here, X and Y are binary series of symptoms. H(X) is the entropy of X and H(X|Y) is the conditional entropy. Entropy can be understood as the variability of X, for example, X = 1111111 or X = 0000000 means that X is regular therefore the entropy (variability) is zero. Mutual information can be also formulated in terms of probabilities, where p(*x*,*y*) is the joint probability of X and Y and p(*x*) is the marginal probability of X, and p(*y*) is the marginal probability of Y. Mutual information is traditionally measured in bits, always positive and limited to the value of the entropy.^31^ Therefore, the highest value that the mutual information can achieve is the value of entropy. Therefore, if the entropy is lower than 1, then the maximum possible correlation using mutual information will also be lower than 1. If the entropy is zero then mutual information will also be zero. This is a main difference from traditional correlation measures, such as Pearson's correlation coefficient where the highest value is always 1 and values of correlation can be positive or negative. Mutual information was chosen because it is suitable for naturally discrete binary data, it is nonlinear and, therefore, able to capture more complex interactions^32^.

Significance of the links is tested using surrogate data testing.^32^, ^33^ For that, a surrogate null model data set is generated using randomized data, where mutual information is calculated using the surrogate data (MI_surr_).^32^ To create the null model, the process of calculating the mutual information with a randomized symptom series is repeated n times (n = 5000). The *P*‐value is estimated by comparing the proportion of MI_surr_ values in the surrogate null model that are larger than the MI obtained from the real data set.^32^ Only statistically significant links after the false‐discovery‐rate correction were kept in the network. In addition, our approach is data‐driven, which is crucial given that no a priori information from Tourette networks exists and, therefore, the underlying data structure is not known. All statistics were calculated in MATLAB (www.mathworks.com).

### Network Measures

We selected measures that can be interpreted in a clinical context. Core‐periphery structure reveals subdivisions of the network, that is, which symptoms are at the center of the network and which symptoms are at its' periphery. The underlying rationale for this graph measure is that a network has a cohesive subgroup densely connected and another subgroup more loosely connected to the core. This does not imply importance of those symptoms, but rather, how well they are interconnected in the network. Degree shows how many connections a symptom has, and which are the most connected. Strength reveals which symptoms are more strongly connected to others, and betweenness centrality is the fraction of all shortest paths that pass through a node. Shortest path is the minimum number of links (steps) to go from one node to another. Betweenness centrality reveals, which symptoms could potentially “hit” or “activate” most symptoms in the network because betweenness is related to shortest path. Taking all these characteristics together helps us to understand which symptoms play a central role in the network. Additionally, we calculated modularity to detect subgroups with similar properties.

We used the brain connectivity toolbox (BCT) (https://sites.google.com/site/bctnet/?pli=1)^34^ for data analysis applied to weighted networks. For more details in how the graph measures are calculated, refer to Data S1 and to the list of measures menu of BCT toolbox (https://sites.google.com/site/bctnet/list-of-measures?authuser=0).

### Data Sharing

The data cannot be openly shared because it was extracted from medical records across a period of 10 years where no permission was collected from patients to pass on their data.

The code is fully available on request to C.G.F.

## Results

### Children and Adolescents

**FIG. 1:**
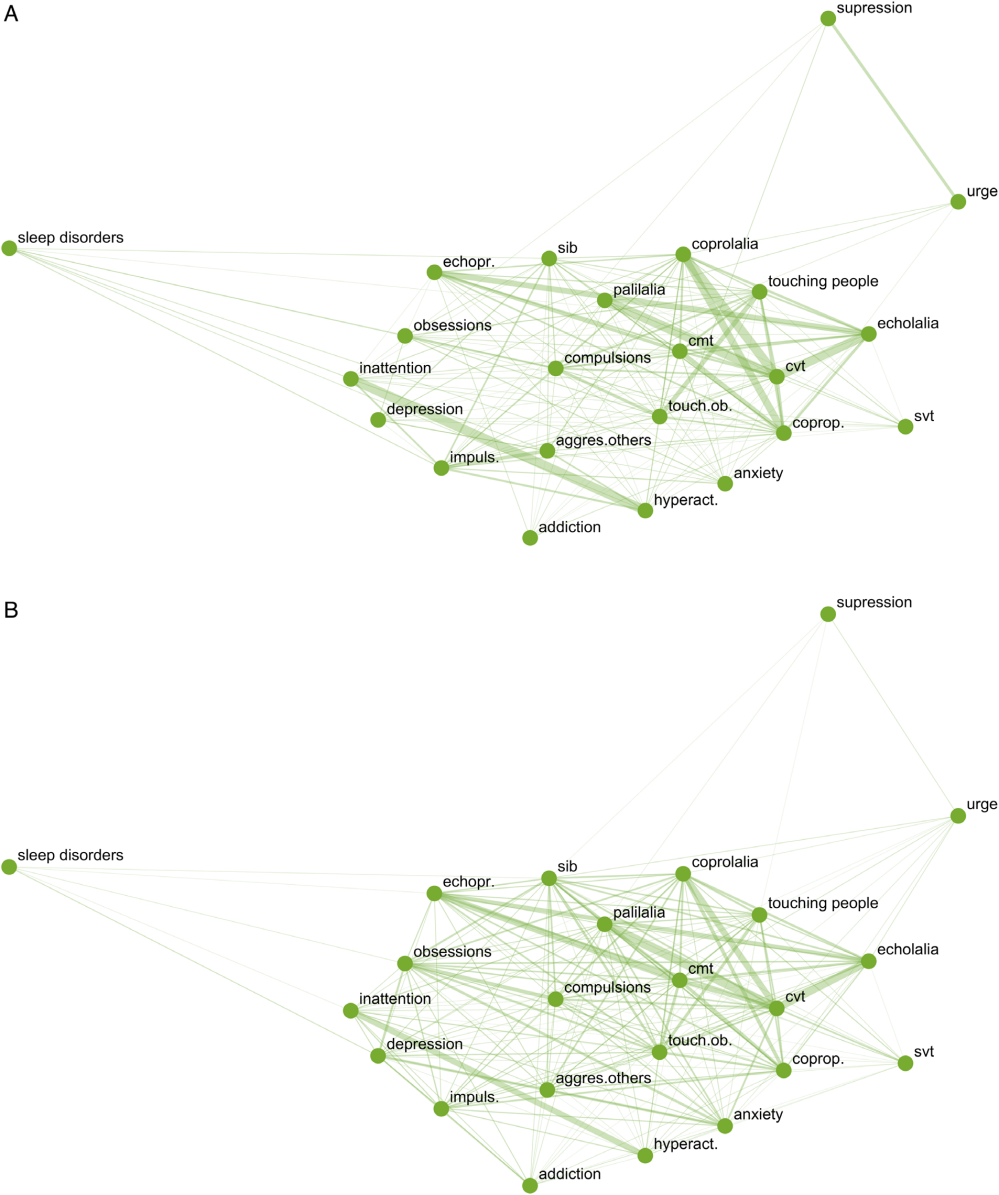
The graph shows the connections among nodes (variables) in the network and the strength of those connections (broader lines represent stronger connections) between the nodes of the network (**A**) in children and adolescents with a chronic tic disorder and (**B**) in adults with a chronic tic disorder. Aggres.others, aggression toward others; cmt, complex motor tics; coprop., copropraxia; cvt, complex vocal tics; echopr., echopraxia; hyperact., hyperactivity; impuls., impulsivity; sib, self‐injurious behavior; svt, simple vocal tics; touch.ob., touching objects; urge, premonitory urges.

Core symptoms were complex motor tics, complex vocal tics, coprolalia, copropraxia, palilalia, echolalia, echopraxia, touching objects, touching people, compulsions, hyperactivity, inattention, and impulsivity (Fig. 1A). The strongest connections were found for complex vocal tics with palilalia (MI = 0.23), coprolalia (MI = 0.23), and echolalia (MI = 0.22), as well as between hyperactivity and inattention (MI = 0.23). Strong connections were also found between coprolalia and copropraxia (MI = 0.18), echolalia and echopraxia (MI = 0.14), and complex motor tics with copropraxia (MI = 0.12).

The core symptoms with higher degree (number of connections) were copropraxia, followed by coprolalia, complex vocal tics, and compulsions (Table 1). Of these, complex vocal tics, coprolalia and copropraxia also had high strength (strength of connections to other variables). Other variables with high strength were coprophenomena and echolalia. The highest betweenness centrality (highest number of shortest paths throughout the network) was found for coprolalia, complex vocal tics, copropraxia, and palilalia, followed by hyperactivity, and impulsivity (Table 1).

**TABLE 1:** Network parameters in young people with chronic tic disorders

Variables	No. (%)	Degree	Strength	BC	Modularity	Core‐periphery
Simple motor tics	528 (99.8)	–	–	–	–	–
Simple vocal tics	498 (94.1)	11	0.1	3	1	Periphery
Complex motor tics	275 (52)	15	0.5	32	1	Core
Complex vocal tics	251 (47.4)	19	1.1	163	1	Core
Coprolalia	113 (21.4)	19	0.8	202	1	Core
Copropraxia	80 (15.1)	19	0.8	116	1	Core
Palilalia	157 (29.7)	13	0.4	64	1	Core
Echolalia	130 (24.6)	18	0.7	39	1	Core
Echopraxia	88 (16.6)	12	0.4	10	1	Core
Touching people	126 (23.8)	18	0.5	37	2	Core
Touching objects	193 (36.5)	15	0.5	3	2	Core
Obsessions	105 (19.8)	12	0.2	19	2	Periphery
Compulsions	300 (56.7)	19	0.5	46	2	Core
Anxiety	149 (28.2)	15	0.2	7	4	Periphery
Depression	41 (7.8)	9	0.1	3	4	Periphery
Addiction	3 (0.6)	10	0.1	3	1	Periphery
Urge	296 (56)	5	0.1	32	3	Periphery
Suppression	391 (73.9)	4	0.1	14	3	Periphery
Hyperactivity	153 (28.9)	14	0.5	59	4	Core
Inattention	235 (44.4)	15	0.4	10	4	Core
Self‐injurious behavior	179 (33.8)	17	0.3	3	4	Periphery
Aggression toward others	59 (11.2)	12	0.3	16	4	Periphery
Impulsivity	317 (59.9)	17	0.4	50	4	Core
Sleep disorders	117 (22.1)	8	0.1	3	4	Periphery

The table shows (from left to right) the variables that were considered for the network analysis, the total number (n) and percentage (%) of children/adolescents who exhibited that symptom, the number of connections of each included symptom within the network (degree), the strengths of those connections (strength), how effectively that variable connects other variables in the network (BC = betweenness centrality), which modularity the variable was assigned to (modularity), and whether the variable belonged to the core or the periphery of the network.

Substance use disorder, tic suppression, premonitory urges, simple vocal tics, depression, and sleep disorders had the weakest connections among the peripheral symptoms (Table 1). The lowest betweenness centrality was found for substance use disorder, depression, sleep disorders, and SIB (Table 1). The strongest connection in the peripheral network was found between premonitory urges and suppressibility of tics (MI = 0.08). It is important to call attention to the fact that peripheral symptoms in the network should not be regarded as less important. The occurrence of these in the observed patient population was merely more independent of the manifestation of the other symptoms.

To look for hidden structures in the network, we ran a modularity measure and found four subgroups, characterized by variables within a module or group having a higher number of connections among themselves than between groups (Table 1).

### Adults

Core symptoms were complex motor tics, complex vocal tics, coprolalia, copropraxia, palilalia, echolalia, echopraxia, touching objects, touching people, obsessions and compulsions, anxiety, and SIB (Fig. 1B, Table 2). The strongest connections among core variables were found between complex vocal tics with palilalia (MI = 0.29), echolalia (MI = 0.23), and coprolalia (MI = 0.19), paralleling the findings in young people. Moreover, strong connections were found for complex motor tics with echopraxia (MI = 0.20), for echopraxia with echolalia (MI = 0.13), and for complex motor tics with complex vocal tics (MI = 0.13), as well as for coprolalia with copropraxia (MI = 0.11).

**TABLE 2:** Network parameters in adults with chronic tic disorders

Variables	No. (%)	Degree	Strength	BC	Modularity	Core‐periphery
Simple motor tics	502 (99.8)	–	–	–	–	–
Simple vocal tics	471 (93.6)	15	0.2	3	2	Periphery
Complex motor tics	287 (57.1)	20	1.0	66	3	Core
Complex vocal tics	267 (53.1)	20	1.4	175	2	Core
Coprolalia	132 (26.2)	19	1.0	50	2	Core
Copropraxia	80 (15.9)	20	0.7	25	3	Core
Palilalia	182 (36.2)	20	0.8	57	2	Core
Echolalia	160 (31.8)	17	0.8	28	2	Core
Echopraxia	150 (29.8)	17	0.8	3	3	Core
Touching people	106 (21.1)	21	0.6	3	3	Core
Touching objects	211 (41.9)	20	0.8	30	3	Core
Obsessions	252 (50.1)	19	0.8	53	1	Core
Compulsions	405 (80.5)	18	0.6	3	1	Core
Anxiety	174 (34.6)	19	0.6	50	1	Core
Depression	195 (38.8)	18	0.5	68	1	Periphery
Addiction	91 (18.1)	16	0.2	3	1	Periphery
Urge	404 (80.3)	9	0.1	34	3	Periphery
Suppression	462 (91.8)	4	0.0	3	3	Periphery
Hyperactivity	138 (27.4)	18	0.4	3	1	Periphery
Inattention	170 (33.8)	18	0.5	48	1	Periphery
Self‐injurious behavior	226 (44.9)	22	0.8	32	3	Core
Aggression toward others	50 (9.9)	19	0.4	3	1	Periphery
Impulsivity	277 (55.1)	18	0.5	5	1	Periphery
Sleep disorders	156 (31)	5	0.1	2	1	Periphery

The table shows (from left to right) the variables that were considered for the network analysis, the total number (n) and percentage (%) of adults who exhibited that symptom, the number of connections of each included symptom within the network (degree), the strengths of those connections (strength), how effectively that variable connects other variables in the network (BC = betweenness centrality), which modularity the variable was assigned to (modularity), and whether the variable belonged to the core or the periphery of the network.

The core symptoms with higher degree (number of connections) were complex motor tics, complex vocal tics, copropraxia, palilalia, SIB, touching people, and compulsions. Of these, complex vocal tics had the highest strength (strength of connections to other variables). Other variables with high strength were complex motor tics and coprolalia. The highest betweenness centrality in the CTD network was found for complex vocal tics, followed by complex motor tics, and depression.

Regarding peripheral variables (Table 2), tic suppression ability and sleep disorder had the least connections in the CTD network. Tic suppressibility was connected only to SIB, premonitory urges, palilalia, and touching people, it had the lowest degree centrality, and the strength was weak. Sleep disorder was connected to inattention, depression, obsessions, echopraxia, and SIB. The strongest connections among periphery variables were between hyperactivity and inattention (MI = 0.18), and between anxiety and depression (MI = 08). The highest betweenness centrality in the periphery group was found for depression, inattention, and premonitory urges. The modularity measure found three subgroups (Table 2).

## Discussion

The network analysis revealed that complex symptoms in TS, including echolalia, palilalia, and coprolalia, are highly interrelated in both adults and children and constituted the core of the CTD network. Previous studies, using hierarchical clustering or factor analysis, have also found that complex tic symptoms cluster together.^21^, ^22^ Although simple motor tics are the most common symptom in CTD, we had to exclude them from this analysis because almost all patients had simple motor tics, and the variable would, therefore, have been located at the periphery of the network, because of its' inability to differentiate between patients. Interestingly, simple vocal tics were also in the periphery and were connected to relatively few other symptoms. Our results are in line with the assumption that there is a severity spectrum in CTD, ranging from simple tics without comorbid symptoms, to complex tics associated with complex phenomena such as echophenomena and coprophenomena and psychiatric comorbidities, but our analysis cannot provide direct proof for this assumption.^6^

Both in young people and adults, complex vocal tics had very high betweenness centrality, the highest strength, and one of the highest degree values. The results show that complex vocal tics are connected to most other symptoms included in this analysis and are involved in most of the shortest paths, connecting symptoms, and comorbidities with each other. Theoretically, this makes complex vocal tics an ideal candidate for therapeutic interventions because the interventions may have knock‐on effects regarding other symptoms and comorbidities. In addition to complex vocal tics, coprophenomena were central symptoms in the young CTD network. These symptoms appear to later shape into complex vocal tics becoming the most central symptom in the adult network. However, causal relationships cannot be inferred from our results because they are correlational in nature.

Interestingly, there were strong connections between similar complex symptoms in young and adult patients, for instance between complex motor tics and complex vocal tics, between coprolalia and copropraxia, echolalia and echopraxia. These symptoms appear to be highly related, irrespective of whether they are motor or vocal phenomena. This result implies that the differentiation between motor and vocal tics in CTDs may be somewhat arbitrary. Future classification systems, therefore, may consider abolishing this differentiation. This suggestion is further supported by the fact that chronic motor tic disorder can be regarded as a milder variant of TS.^6^

In children and adolescents, core symptoms comprised mostly complex CTD symptoms (complex motor tics, complex vocal tics, coprolalia, copropraxia, palilalia, echolalia, and echopraxia), touching people or objects, compulsions, but not obsessions. Core symptoms also included inattention, hyperactivity, and impulsivity, indicating that symptoms of ADHD are closely linked to CTD in childhood. Moreover, ADHD symptoms were not only part of the core network in young people, they had high betweenness centrality, indicating that many of the nodes in the network are effectively connected through ADHD symptoms in children and adolescents. It is possible that when ADHD treated in childhood, its' treatment may positively affect other symptoms.

In contrast, ADHD‐related symptoms were not part of the core network in adults, but obsessions and compulsions were. Previous research in adults has shown that complex CTD symptoms commonly cluster with OCD.^22^ The results are in line with studies showing a close relationship between CTD and OCD in adulthood^35^, ^36^, ^37^ and provide further support for the assumption of a TS‐OCD spectrum in adults.^38^ Longitudinal research has shown that ADHD typically starts even before tic onset, whereas OCD has a later childhood onset, and depression and anxiety disorders may follow later in childhood or adolescence and early adulthood.^39^

In young people and adults, suppressibility of tics and premonitory urges only had few and weak connections, however, particularly premonitory urges were still important to the CTD network in terms of shortest paths, that is, connecting other symptoms effectively. Whether premonitory urges are a prerequisite for tic suppression has been debated in the literature, with some evidence against this assumption.^14^, ^15^ Our results suggest that premonitory urges are connected to the ability to suppress tics in children, but less so in adults. This could indicate that urges and suppression ability are related, albeit not a prerequisite, and that they grow more closely associated with other CTD symptoms in adult patients. It is possible that young patients who are good at using premonitory urges to suppress their tics are more likely tic‐free in adulthood. In addition, it is interesting that the ability to suppress tics was unrelated to most other variables in adults, such as impulsivity^19^ or inattention.^15^ To date, it is largely unclear why some patients are better at suppressing their tics than others.^20^

In adults, depression, anxiety, substance use disorder, and sleep disorder were at the periphery. Apart from OCD and ADHD, depression and anxiety are the most common comorbidities in TS,^40^ but do not appear to be as intimately related to CTD as ADHD and OCD. This is in line with studies showing strong genetic connections between TS, ADHD, and OCD.^36^, ^41^ Additionally, this is in line with recent research, showing that children with comorbid ADHD and OCD were more likely to show depressive symptoms, and these moderated higher functional impairment.^42^ It would be interesting to see at what age relationships to depression, sleep disorders, and particularly SIB start to emerge. Our data suggests that anxiety and depression were mainly related to CTD via OCD in this sample, whereas sleep disorders are more closely associated with comorbid ADHD than with CTD directly.

It is particularly interesting that SIB did not play a significant role in the symptom network in young people, but became a central symptom in adults with chronic tic disorders. SIB was closely connected with complex motor and vocal tic phenomenology, and this is in line with a recent study, also showing the very close connection between complex tics and SIB.^5^ The lifetime prevalence of self‐injurious behavior in patients with CTD is estimated to be quite high (~40%),^4^ with up to 84% of patients reporting having experienced an urge to perform auto‐aggressive acts.^5^ Previous research in adults with CTD suggests that SIB is correlated with tic severity^4^ and comorbidities.^43^ SIB in CTD might be very similar to complex tics because they are perceived as involuntary, patients try to stop them,^44^ and they can be accompanied by an urge.^45^

The modularity analysis showed that symptom clusters were more clearly distinct in young people (ie, symptoms related to CTD clustered in one group) except premonitory urges and suppressibility of tics, which formed a separate group. Symptoms of OCD, including obsessions, compulsions, touching people or objects, anxiety, depression, and SIB formed a distinct cluster, and so did symptoms of ADHD, together with aggression toward others and sleep disorders. It is interesting that aggression toward others (which is not considered a TS symptom)^46^ was related closely to symptoms of ADHD, whereas SIB was closely linked with symptoms of OCD and that SIB and obsessions moved into the core symptom cluster in adults.

In adults, results showed a group consisting of vocal symptoms (ie, simple vocal tics, complex vocal tics, coprolalia, palilalia, and echolalia). A second group consisted of TS‐related motor symptoms and compulsion‐like symptoms (ie, complex motor tics, copropraxia, echopraxia, touching people, touching objects, premonitory urges, tic suppression, and SIB). The third group included all comorbidities that were included in the network analysis. Despite the division of motor and vocal symptoms into separate modules, it should not be concluded that they are qualitatively different. On the contrary, the analysis of the strength of connections indicates that motor and vocal symptoms are highly related. However, it is plausible that if a patient has complex vocal tics, some of those may include coprolalia, Palilalia, or echolalia, and therefore, vocal symptoms are necessarily related to each other.

Regarding clinical implications, it might be worth studying whether the treatment of symptoms as complex motor and vocal tics, and comorbid obsessions might lead to reductions in other symptoms in adults. The analyses presented here rely on associations, therefore, we cannot assume that treating one symptom will directly affect another symptom. However, this should be explored. Although compulsions had high strength (ie, were strongly connected to other symptoms) obsessions were most efficiently connected to other symptoms. It is possible that this is the case because compulsions are often driven by obsessions and can, therefore, be treated by treating obsessions.

### Strengths and Limitations

A strength of the study is the large cohort of patients that were diagnosed by a TS/CTD expert. Limitations include a limited number of comorbidities, which were assessed by a clinician according to diagnostic criteria, not standardized assessments, the fact that data was collected during a 10‐year period, and spanning several versions of the DSM. However, core criteria for the disorders included here remained the same across those versions. Another limitation is that simple tics were not differentiated. Differentiating simple tics might lead to a better integration into the network, however, differentiating between all simple tics would have led to more than 100 different tics categories here and was, therefore, not possible.

### Conclusions

Core symptom networks in young and adult patients with CTD included mainly complex tics, and tic‐related phenomena, such as echophenomena, paliphenomena, and coprophenomena. Core symptoms in childhood included symptoms of ADHD, whereas core symptoms in adults included symptoms of OCD instead. Interestingly, SIB was a central symptom in adults, but not children, with CDTs. Our networks suggest that a differentiation between motor and vocal tics is somewhat arbitrary, because this division is not reflected in the networks.

## Author Roles

(1) Research Project: A. Conception, B. Organization, C. Execution; (2) Statistical Analysis: A. Design, B. Execution, C. Review and Critique; (3) Manuscript: A. Writing of the First Draft, B. Review and Critique.

C.G.F.: 1B, 1C, 2A, 2B, 2C, 3A, 3B

V.B.: 1A, 1C, 2A, 2B, 2C, 3A, 3B

E.J.: 1A, 2A, 2B, 2C, 3B

C.G.: 1A, 1B, 2A, 2C, 3B

S.K.: 1A, 1B, 2A, 2C, 3B

K.M.: 1A, 1B, 2A, 2C, 3B

## Disclosures

**Ethical Compliance Statement:** This secondary data analysis of the dataset presented in Sambrani et al,^9^ therefore, no written informed consent was neither required nor needed ethics committee approval. We confirm that we have read the Journal's position on issues involved in ethical publication and affirm that this work is consistent with those guidelines.

**Funding Sources and Conflicts of Interest:** No specific funding was received for this work. The authors declare that there are no conflicts of interest relevant to this work.

**Financial Disclosures for Previous 12 Months:** C.G.F. was supported by the Deutsche Forschungsgemeinschaft (DFG, German Research Foundation) – 178316478 – C7 and ERC‐2022‐CoG‐BrainScape‐101086188. V.B. was supported by the Academy of Medical Sciences (Springboard grant). C.G. holds the Wolf Chair for Neurodevelopmental Psychiatry, a joint Hospital‐University Named Chair between the University of Toronto, UHN, and the UHN Foundation. He received honoraria from the Movement Disorder Society for educational activities and academic research support from the VolkswagenStiftung (Freigeist Fellowship). E.J. has nothing to declare. S.K. was supported by GRK 2753/1–449640848, ERC‐2022‐CoG‐BrainScape‐101086188, SFB 936‐178316478, TRR 169/C8. K.M. declares consultancies for Canymed, Emalex, Eurox Group, Sanity Group, Stadapharm, Swiss alpinapharm, Synendos Therapeutics AG, Tetrapharm, and Triaspharm; is on the Advisory Boards of Branchenverband Cannabiswirtschaft e.V. (BvCW), Sanity Group, Synendos Therapeutics AG, Syqe Medical, and Therapix Biosciences; has received honoraria from Almirall, Bundesverband der pharmazeutischen Cannabinoidunternehmen (BPC), Cogitando, Emalex, Grow, Medizinischer Dienst Westfalen Lippe, Noema, streamedup!, and Vidal; receives royalties from Elsevier, Medizinisch Wissenschaftliche Verlagsgesellschaft Berlin, and Kohlhammer; receives grants from DFG: GZ MU 1527/3–1 and GZ MU 1527/3–2 and Almirall Hermal. K.M. is the associate editor for “Cannabis and Cannabinoid Research,” an Editorial Board Member of “Medical Cannabis and Cannabinoids,” and “MDPI‐Reports,” and a Scientific board member for “Zeitschrift für Allgemeinmedizin.”

## Supporting information


**Data S1.** Network methods and graph measures.

## Data Availability

The data cannot be openly shared because it was extracted from medical records across a period of 10 years where no permission was collected from patients to pass on their data.The code is fully available upon request to CGF.
