# Supplementary material for: Symptom Network Analysis in a Large Sample of Children and Adults with a Chronic Tic Disorder
Source: Mov Disord Clin Pract. 2024 Jul 25;11(10):1232–40. doi: 10.1002/mdc3.14167 (PMC11489602; doi:10.1002/mdc3.14167)
Supplement: Supplementary file 1 — Data S1. Network methods and graph measures. [file MDC3-11-1232-s001.docx]

**Symptom network analysis in a large sample of children and adults with a chronic tic disorder**

Caroline Garcia Forlim, PhD^1,2^*, Valerie Brandt, PhD^3,4^*, Ewgeni Jakubovski, PhD^4^, Christos Ganos, PhD^5^, Simone Kühn, PhD^1,2^*, Kirsten Müller-Vahl, PhD^4^*

*these authors contributed equally to the work

^1^Neuronal Plasticity Working Group, Department of Psychiatry and Psychotherapy, University Medical Center Hamburg-Eppendorf, Martinistraße 52, 20246, Hamburg, Germany

^2^Lise Meitner Group for Environmental Neuroscience, Max Planck Institute for Human Development, Lentzeallee 94, 14195, Berlin, Germany

^3^ School of Psychology, Centre for Innovation in Mental health, University of Southampton, Southampton, UK
^4^ Clinic of Psychiatry, Social Psychiatry and Psychotherapy, Hannover Medical School, Hanover, Germany

^5^ Movement Disorder Clinic, Edmond J. Safra Program in Parkinson’s Disease, Division of Neurology, University of Toronto, Toronto Western Hospital, Toronto, Ontario, Canada

**Supplementary Material**

*Network Methods*

As our data is naturally discrete and binary, we used the simple estimation of mutual information:


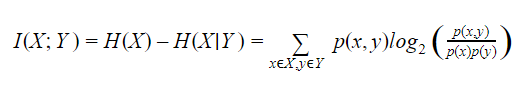


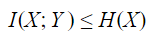


where I(X;Y) is the mutual information between X and Y. Here X and Y are binary series of symptoms. H(X) is the entropy of X and H(X|Y) is the conditional entropy.

Entropy can be understood as the variability of X, for example, X=1111111 or X=0000000 means that X is regular therefore the entropy (variability) is zero. Mutual information can be also formulated in terms of probabilities, where p(x,y) is the joint probability of X and Y and p(x) is the marginal probability of X, and p(y) is the marginal probability of Y. Marginal probability is the simple probability of occurrence, that is, number of times that 0 or 1 appears divided by the total size of the series, e.g. in series x=001001 the marginal probability is Px(0)=4/6 and Px(1)=2/6. The joint probability requires two series e.g. x=001001 and y=001110 and is the probability that a value in x and a value in y occur simultaneously. For that a matrix is computed, where we count how many times x=0 and y=0 simultaneously occur, followed by how many times x=0 and y=1 occur together, etc and then divide by the total size of the series.

Mutual information is traditionally measured in bits, always positive and limited to the value of the entropy. Therefore, the highest value that the mutual information can achieve is the value of entropy. This is a main difference from traditional correlation measures, such as Pearson’s correlation coefficient where the highest value is always 1 and values of correlation can be positive or negative. Thus, if the entropy is lower than 1, then the maximum possible correlation using mutual information will also be lower than 1. If the entropy is zero then mutual information will also be zero.

In our case, the maximum entropy value is 1 and this is possible when a probability of 0 is 50% and of 1 is also 50%. All other probability combination will lead to values of entropy lower than 1 reaching the extreme case of entropy equals 0 when having 100% probability of 0 or 1.

Mutual information was chosen because it is suitable for naturally discrete binary data, it is nonlinear and therefore able to capture more complex interactions.

^1^The conditional entropy H(Y|X) is the concept of entropy for two variables and informs us about the amount of information needed to describe Y given that X is known, or we can think also in terms of variability, in this case, the conditional entropy H(Y|X) is the variability of Y given that X is fixed.

*Networks Measures*

These measures are defined as: a) Degree – the node degree is the total number of links connected to a particular node; b) Strength - the node strength is the sum of the weights from all links connected to the node; c) Betweenness - the fraction of all shortest paths that pass through a node. Shortest path is the minimum number of links to go from one node to another; d) Core-periphery – core/periphery partitions the network into two groups of nodes: a core and a periphery group; The underlying rationale for this graph measure is that a network has a cohesive subgroup densely connected and another subgroup more loosely connected to the core. The definition of the core-periphery structure according to the BCT toolbox is “The core/periphery subdivision is a partition of the network into two non-overlapping groups of nodes, a core group and a periphery group, in a way that maximizes the number/weight of within core-group edges, and minimizes the number/weight of within periphery-group edges.” More details of the formalization and algorithms can be found in (Borgatti & Everett, 2000)^1^ e) Modules – subdivision of networks in groups of nodes which have a high number of within-modules connections and a low number of between module connections, here we will refer modules as groups. For more details on the network measures, their definition and how they are calculated please refer to the list of measures menu of BCT toolbox (https://sites.google.com/site/bctnet/list-of-measures?authuser=0).

**Refererences**

1. Borgatti SP, Everett MG. Models of core/periphery structures. *Social Networks.* 2000;21:375-395.
